# Recurrent Kawasaki Disease: A Case Report of Three Separate Episodes at >4-Year Intervals

**DOI:** 10.3390/children5110155

**Published:** 2018-11-21

**Authors:** Nikita Goswami, Katherine Marzan, Elizabeth De Oliveira, Sharon Wagner-Lees, Jacqueline Szmuszkovicz

**Affiliations:** 1 CHLA Pediatric Rheumatology, Children’s Hospital Los Angeles, 4650 Sunset Blvd, Mailstop #60, Los Angeles, CA 90027, USA; kmarzan@chla.usc.edu; 2 Pacific Pediatric Cardiology, Pacific Pediatric Cardiology Medical Group, Pasadena, CA 91105, USA; pacpedscard@gmail.com; 3 CHLA Pediatric Cardiology, Children’s Hospital Los Angeles, 4650 Sunset Blvd, Los Angeles, CA 90027, USA; SWagnerLees@chla.usc.edu (S.W.-L.); JSzmuszkovicz@chla.usc.edu (J.S.)

**Keywords:** Kawasaki disease, coronary aneurysm, atypical, recurrence

## Abstract

Kawasaki disease (KD) is a self-limited systemic vasculitis, most often occurring in children 1–5 years old. It has a 2% recurrence rate and is associated with coronary aneurysms (CA), which can develop within two weeks of onset. A 25% increased risk is noted in patients who are recalcitrant to treatment. We describe a patient with recurrence of KD three times, approximately four years apart. A 10-year-old female with two previous episodes of KD, at 11 months and five years of age), in which she met five out of five criteria for KD and had no coronary involvement, presented with 15 days of fever, conjunctivitis and mucocutaneous changes. Infectious work-up was negative, and she was diagnosed with incomplete KD meeting three out of five criteria. An echocardiogram (ECHO) on day 12 revealed dilation of the right coronary artery (RCA) and left coronary artery (LCA). Treatment with intravenous immunoglobulin (IVIG) and high-dose aspirin was started at an outside hospital. After transfer, serial ECHOs showed evolving coronary aneurysms, left anterior descending (LAD) z-score + 8.2 and RCA z-score + 4.0. She received 10 mg/kg infliximab (day 18) and began clopidogrel. A cardiac MRI (day 20) demonstrated progression of the LAD aneurysm, with a z-score + 13, and warfarin was started. To our knowledge, this is the first report of recurrent KD occurring three times at ~5 year intervals.

## 1. Introduction

Kawasaki disease (KD) is a self-limited systemic vasculitis, typically occurring in children 1–5 years of age. The exact etiology of KD is not known. While there is data to support an infectious trigger in a genetically predisposed individual, no specific infectious triggers or definitive genetic markers have been clearly identified [1].

The diagnosis of complete KD is based on the presence of ≥5 days of fever and four of the five principal clinical features. These features include bilateral bulbar conjunctivitis without exudate, erythema and cracking of lips and/or strawberry tongue, rash (maculopapular or diffuse erythroderma), hand and foot erythema and edema, and unilateral cervical lymphadenopathy [2]. KD has a 2% recurrence rate, and recurrence is usually within the first year [2]. Generally, coronary aneurysms develop within two weeks of disease onset and are diagnosed with an ECHO. Coronary changes (z-score ≥ 2.5) can be seen in these patients, most commonly occurring in the left anterior descending (LAD) or right coronary artery. The development of coronary aneurysms raises clinical suspicion for the diagnosis of KD.

Patients with incomplete or atypical KD do not present with all the classic clinical features. Individuals with incomplete KD have delayed diagnosis and an increased risk of developing coronary artery abnormalities. Prolonged fever is sometimes the exclusive clinical finding in an atypical presentation, while some may have subtle clinical findings, such as a rash or conjunctivitis that is fleeting at the time of presentation. Because of the fluctuating symptoms, the history is a critical part of the diagnostic algorithm. Secondary to delays in diagnosis, these atypical cases have a 25% increased risk of developing coronary aneurysms [2]. In addition, these cases may be refractory to treatment due to the progression of disease prior to diagnosis.

This case illustrates that although KD occurs more often in infants and toddlers, it can occur later in life, and with the recurrence of disease one can have new coronary changes.

## 2. Case Summary

A ten-year-old female presented with 10 days of fever, nonpurulent conjunctivitis and mucocutaneous changes (strawberry tongue and cracked lips) with a negative infectious workup, leading to a diagnosis of incomplete KD meeting three out of five clinical criteria. She had previously had classic KD, meeting five out of five clinical criteria, on two previous occasions. These occurred at 11 months and five years of age. No coronary abnormalities were identified by ECHO during the first two episodes. She was followed by a cardiologist until one year after her second episode, at which time she was discharged from cardiology follow-up. During her third episode of KD, an ECHO was performed on day 12 of the fever, which revealed dilation of the right coronary artery (RCA) and left coronary artery (LCA). She was then treated with IVIG (2 g/kg) and high-dose aspirin (ASA) at the outside hospital, on day 13 of illness. She was transferred to our institution on day 14.

Upon arrival to our institution she did not have hypertension, fever, or tachypnea and was saturating well on room air. Her exam was unremarkable, with normal cardiac rate and rhythm, clear lungs, and normal bowel sounds without any distention, tenderness or organomegaly. On examination, she had no rash, and had full range of motion of all joints without effusions.

**Figure 1:**
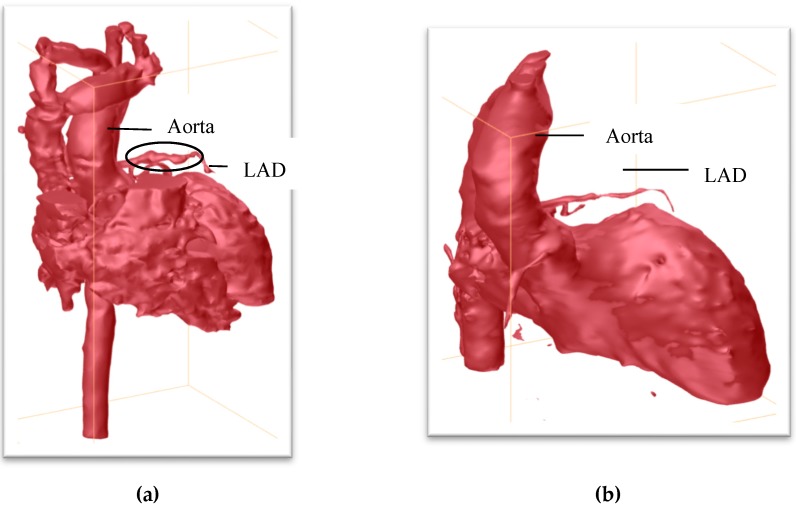
Comparison of initial cardiac MRI upon presentation and repeat cardiac MRI about 8 months later. (**a**) Three-dimensional cardiac MRI. This image demonstrates the aneurysm of left anterior descending coronary artery (LAD) which is classified as a giant aneurysm with a z-score of 13. The circle shows the beading of the LAD secondary to aneurysms. (**b**) Repeat MRI showing improvement of the LAD.

Her initial labs upon arrival showed evidence of thrombocytosis, acute inflammation (CRP 3.2), and normal cardiac function, and normal troponin and BNP. She did not demonstrate features of macrophage activating syndrome/secondary hemophagocytic lymphohistiocytosis and had normal ferritin, fibrinogen, and triglycerides. Her elevated ESR was expected status post IVIG infusion. The infection workup for tuberculosis, cytomegalovirus, Epstein–Barr virus, human herpesvirus 6, parvovirus B19, and respiratory viral panel were all negative. After initial evaluation, multiple serial ECHOs (every 1–2 days) were performed, which showed evolving coronary ectasia, with a left anterior descending artery (LAD) z-score + 8.2 and RCA z-score + 4.0. She received 10 mg/kg infliximab on day 18 of illness, and began clopidogrel (in addition to aspirin). Because of concern regarding the enlarged proximal coronaries and difficulty visualizing her distal arteries, a cardiac MRI was performed. These images demonstrated a giant aneurysm of the distal LAD, z-score + 13.3 (Figure 1). The location of this aneurysm was difficult to visualize by ECHO because of its distal position. After diagnosis of a giant coronary aneurysm, anticoagulation was altered to include heparin, as a bridge to therapeutic warfarin doses.

Given our patient’s atypical KD presentation at an older age, it was important to consider other etiologies. Takayasu arteritis was ruled out, as she had normal four-extremity blood pressures and a normal MRA of the chest, abdomen and pelvis. In addition, she had no symptoms of hypertension, rash, neuropathy, muscle involvement, or renal involvement to suggest polyarteritis nodosa (PAN). An interventional radiology angiogram during her acute illness was not performed because of her lack of PAN systemic symptoms. Furthermore, her response to infliximab therapy with resolution of KD stigmata/fever was reassuring, and subsequent improvement was noted in her coronary abnormalities over time. A repeat cardiac MRI (Figure 1) nine months later showed resolution of the coronary aneurysms and lingering ectasia in RCA and left main coronary artery (LMCA), with vessel wall thickening present in both. She was weaned off warfarin and transitioned to aspirin and Plavix.

## 3. Discussion

To our knowledge, this is the first report of recurrent KD occurring three times with episodes after prolonged intervals of four and five years. Our patient had three distinct episodes of KD: the first two presented as typical cases without the development of coronary aneurysm and the most recent as atypical, with coronary involvement and a giant aneurysm at three weeks. Her older age and atypical presentation led to a delay in diagnosis, as she was initially thought to have a viral illness and sent home. IVIG was not initiated until day 13 of illness. The differential diagnoses included Takayasu arteritis (TA) and polyarteritis nodosa (PAN). TA was excluded based on normal four-extremity blood pressures and a normal MRA of the chest, abdomen and pelvis. The question of polyarteritis nodosa (PAN) was more complex since PAN and KD share clinical manifestations that make them difficult to differentiate [3]. PAN is a disseminated vasculitis of unknown etiology of the small and medium vessels with multiorgan involvement. However, KD is a self-limited vasculitis with cardiac, skin and eye manifestations. In a few cases, GI issues (obstruction and ileus) and renal manifestations (renal artery stenosis and fibrinoid degeneration of renal arteries) can be seen in cases of KD [3]. Further imaging of vessels generally detects multiple aneurysms as well as multiple organ system involvement in patients with PAN [3]. An MRI with angiography of the neck, chest and abdomen did not show aneurysms in other vessels. She lacked evidence of hypertension, vasculitic rash, myositis, abdominal pain, renal involvement, or neuropathy to support PAN. An IR angiogram is considered the gold standard test but was not performed secondary to the lack of PAN-associated systemic symptoms and response to the Infliximab therapy with rapid resolution of KD stigmata after IVIG, and the improvement of coronary ectasia over time.

Interestingly, our patient’s recurrent KD occurred at an older-than-expected age and at four and nine years after the initial diagnosis, rather than within the first year. Recurrent KD more than two years from diagnosis is rare. However, there has been a case report of recurrence 19 years after initial diagnosis [4], as well as reports of recurrent KD from two months to 23 years of age [4,5]. In addition, multiple recurrences can be seen in KD [6]. One case report describes it recurring four times within a 33 month period in contrast to the longer intervals between recurrences seen in our patient [7]. Initial treatment for KD is high dose ASA followed by IVIG 2 g/kg. Early and late recurrent fevers can be seen post IVIG (24–72 h and >72 h respectively) and can resolve without additional treatment (Yoshida et al.). However, if patients remain refractory to treatment then some alternatives are to administer Infliximab or a second dose of IVIG and evaluate for response. Recurrent KD is defined as a repeat episode of complete or incomplete KD that occurs after the complete resolution of the previous episode. These patients should receive standard therapy [2]. The fevers that occur with recurrence require further evaluation with an ECHO due to concern for the evolution of coronary ectasias [8].

KD patients with recurrent episodes are more likely to be older, have atypical presentations, and coronary artery abnormalities regardless of IVIG therapy [9]. Aneurysms were only identified in our patient with the third episode. *Z*-scores are a measurement tool used to detect the standard deviation from normal for age per weight and height. If patients with typical KD are adequately treated within 10 days of presentation then there can be increased z-scores for approximately five weeks after initial presentation; but the prevalence of coronary aneurysms is low [10]. In typical KD, an ECHO is performed in the hospital at the time of diagnosis, followed by a repeat ECHO within two weeks. Further ECHOs are performed after more time has elapsed to assess the improvement or progression around 6–8 weeks [2]. ECHOs were performed on our patient on admission (day 13) and more frequently than routine due to rapid progression and severity of ectasia. A cardiac MRI detected a distal giant aneurysm of the LAD with a z-score up to 13 (on previous ECHO z-score 8.2) that was not clearly evident on the ECHO.

Alternative therapies for patients resistant to IVIG and high dose aspirin include anti-tumor necrosis factor agents. Infliximab, a monoclonal antibody to TNFα is a rescue therapy as well as a primary therapy. Tremoulet et al. showed that patients refractory to IVIG had significantly decreased z-scores of LAD compared to the IVIG group at two weeks. However, the difference at five weeks between the treatment group was equivalent [11].

## 4. Conclusions

Although KD primarily affects infants and toddlers, this is an example of an adolescent experiencing KD more than four years from her previous episodes. The symptoms in an adolescent may be fleeting or atypical (meeting less than four of the five criteria), which can result in delayed diagnosis. A delay in diagnosis increases the likelihood of developing coronary artery aneurysms [9]. Our patient experienced KD three times, interestingly with coronary involvement in the form of a giant aneurysm only noted with the most recent episode. It is possible that the first two episodes had coronary involvement that was undetected by ECHO. Additional imaging with a CT angiogram (CTA) or cardiac MRI can be critical in delineating the anatomy and determining the extent of the vessel involvement in KD. Carbone et al. noted that CTA was equal to an invasive IR angiogram in monitoring coronary aneurysms and their progression [12]. Cardiac MRIs have been shown to be an effective modality to assess the systolic function of left and right ventricles, and to evaluate for ischemic changes after the diagnosis of KD [13]. If there is uncertainty about the progression of proximal coronary ectasias then it may be beneficial to obtain a cardiac MRI or CTA to further investigate coronary involvement, as it can impact the medical management. Regardless of age, it is always important to have KD on the differential in a patient with a fever of unknown origin.
